# Supplementary material for: All-at-once RNA folding with 3D motif prediction framed by evolutionary information
Source: Nat Methods. 2025 Oct 3;22(10):2094–106. doi: 10.1038/s41592-025-02833-w (PMC12510878; doi:10.1038/s41592-025-02833-w)
Supplement: Supplementary file 3 — Source code to run CaCoFold-R3D. Data and results. [file 41592_2025_2833_MOESM3_ESM.gz › supplemental_material/results/CaCoFold-R3D/CaCoFold-R3D_Rfam/RF01981_PCGEM1.R2R.sto.pdf]

RF01981_PCGEM1

5′-R A A A C A A A G G U G C C U U U G C C A A U A U U R U G U U U Y ● Y U U U G A C A A G Y Y Y U G A G A U U U C U G A G G G G A A U C C A Y U R U A A A U G G G A U C A G G U C A U U C A U U U A U G U U G U G U R C A A A U A U A A U Y U ● A A G A U A C A R C C U Y
